# Hypolipidemic Activity of Amine-Borane Aducts of Cyclohexylamine and Toluidine in Rodents

**DOI:** 10.1155/MBD.1995.221

**Published:** 1995

**Authors:** Bruce S. Burnham, S. Y. Chen, A. Sood, Bernard F. Spielvogel, Iris H. Hall

**Affiliations:** 1 Division of Medicinal Chemistry and Natural Products School of Pharmacy University of North Carolina Chapel Hill N.C. 27599-7360 USA; 2 Boron Biologicals, Inc. 620 Hutton Rd. Raleigh N.C. 27606 USA

## Abstract

The amine-borane adducts of cyclohexylamine and toluidine were observed to be potent hypolipidemic agents in mice, I.P. and rats orally at 8 mg/kg/day lowering both serum cholesterol and triglyceride levels after 14-16 days. These compounds were able to lower tissue lipids including the cholesterol content of the aorta wall. The agents successfully lower VLDL- and LDL-cholesterol content while elevating HDL-cholesterol content significantly. The agents also modulate lipid regulatory enzyme activities in a manner to reduce liver lipid levels. These studies demonstrate that the nitrogen atom does not have to be apart of the aromatic ring as in heterocyclic-amine borane to afford good hypolipidemic activity in rodents.


### HYPOLIPIDEMIC ACTIVITY OF AMINE-BORANE ADUCTS OF CYCLOHEXYLAMINE AND TOLUIDINE IN RODENTS

Bruce S. Burnham[1], S.Y. Chen[1], A. Sood[2], Bernard F. Spielvogel[2] and Iris H. Hall[1]

[1] Division of Medicinal Chemistry and Natural Products, School of Pharmacy, University of North Carolina, Chapel Hill, N.C. 27599-7360, USA
[2] Boron Biologicals, Inc., 620 Hutton Rd., Raleigh, N.C. 27606, USA

ABSTRACT

The amine-borane adducts of cyclohexylamine and toluidine were observed to be potent hypolipidemic agents in mice, I.P. and rats orally at 8 mg/kg/day lowering both serum cholesterol and triglyceride levels after 14-16 days. These compounds were able to lower tissue lipids including the cholesterol content of the aorta wall. The agents successfully lower VLDL- and LDL-cholesterol content while elevating HDL-cholesterol content significantly. The agents also modulate lipid regulatory enzyme activities in a manner to reduce liver lipid levels. These studies demonstrate that the nitrogen atom does not have to be apart of the aromatic ring as in heterocyclic-amine borane to afford good hypolipidemic activity in rodents.

### INTRODUCTION

Previously we have examined a number of amine-cyanoboranes[1], amine-carboxyboranes and their amides and esters[2-5], di- and tri-peptide boranes[6], heterocyclic-amine boranes[7], polyborate salts[8], choline and thiocholine boranes[9], phosphoacetate boranes[10] and deoxyribonucleoside cyanoboranes[11]. These agents were effective in rodents at 8 mg/kg/day, significantly lowering both serum cholesterol and triglyceride levels after 14-16 days. Cholesterol levels were lowered in rat VLDL and LDL fractions while the HDL cholesterol levels were markedly elevated. The mode of action of these derivatives was to acclerate bile cholesterol excretion and alteration of regulatory enzyme activities involved in de novo lipid synthesis. Furthermore, sub-acute toxicity studies in mice indicated no organ specific toxicity and mean survival doses were high[12]. Thus, we have decided to examine a series cyano- and carboxyborane adducts of cyclohexylamine and toluidine for their hypolipidemic activity in rodents. These studies should determine if the nitrogen atom is necessary in the aromatic ring to maintain activity as a hypolipidemic agent in rodents.

### MATERIALS and METHODS

Source of compounds
All of the compounds were synthesized previously and chemically and physically charecterized[13]: cyclohexylamine-cyanoborane 1, cyclohexylamine-carboxyborane 2, N-methylcyclohexylamine-carboxyborane 3, N-ethylcyclohexylamine-carboxyborane 4, N,N-dimethylcyclohexylamine-carboxyboranes 5, 2-methylcyclohexylamine-carboxyborane 6, 3-methylcyclohexylamine-carboxyborane 7, 4-methylcyclohexylamine-carboxyborane 8, N-ethylcyclohexylamine-cyanoborane 9, N-

methylcyclohexylamine-cyanoborane 10, N,N-dimethylcyclohexylamine-cyanoborane 11, 2-methylcyclohexylamine-cyanoborane 12, 3-methylcyclohexylamire-cyanoborane 13, 4- methylcyclohexylammine-cyanoborane 14, 2,3-dimethylcyclohexylamine-cyanoboranes 15, o-toluidine-cyanoborane 16, m-toluidine-cyanoborane 17, p-toluidine-cyanoborane 18, and p-anisidine cyanoborane 19. All isotopes were purchased from New England Nuclear. Substrates and co-factors were obtained from Sigma Chemical Co. Sprague Dawley male rats were obtained from Charles River Laboratory. $CF_1$ mice were obtained from Jackson Laboratory. Animals were maintained in light cycles of 12 h at 22°C. Rats were maintained in individual wire cages and mice were housed three/plastic cage. Food (Agway/Prolab Animal Diet) and water were ad libitum.

Normolipidemic studies

For structure activity studies, $CF_1$ male mice (~28g) were administered amine-borane adducts of cyclohexylamine and toluidine in 1% CMC at 8 mg/kg/day, I.P. Blood samples were obtained on days 9 and 16 between 7:30 and 8:30 a.m. Daily dosing of the agents was between 9:00 and 10:00 a.m. the serum was obtained by centrifuging the blood for 10 min. at 3500 g. The serum cholesterol levels were determined by a modification of the procedure Liebermann-Burchard reaction [14]. Serum triglyceride were determined using a commercial kit [Boehringer Mannheim Diagnostics]. Sprague Dawley male rats (~230 g) were administered orally Compounds 7, 9 or 18 at 8 mg/kg/day, for two weeks. Weekly blood samples were obtained by tail vein bleeding.

Animal weight, organ weight and food consumption

Control and treated normolipidemic Sprague Dawley male rat(~230g) weights were obtained and expressed as a percentage of the initial body weight (week zero). Food consumption (gm/day/rat) was noted for two weeks for control and treated rats[15].

Tissue lipid levels

Normolipidemic Sprague Dawley male rats (~230 g) which were treated orally for two weeks with compound 7, 9 or 18 at 8 mg/kg/day, were sacrificed and tissue samples of the liver, small intestinal mucosa and aorta were removed. A 24 hr fecal sample was also obtained. A 10% homogenate in 0.25 M sucrose + 0.001 M EDTA, pH 7.2, was prepared for each tissue. An aliquot (2 ml) of the homogenate was extracted[16-17] and the number of mg of lipid extracted was weighed. The lipid residue was taken up in methylene chloride and the levels of cholesterol [14], triglycerides , neutral lipids[18] and phospholipids[19] were determined. Protein content of the whole homogenate was determined[20].

Serum lipoprotein fractions

Normolipidemic Sprague Dawley male rats(~230g) treated for two weeks with compounds 7, 9 or 18 at 8 mg/kg/day, orally were anesthesized with ether and blood (~10 ml) was collected from the abdominal vein. Serum was separated from whole blood by centrifugation at 3500 rpm. Aliquots of the serum were separated into chylomicrons, VLDL, HDL and LDL by ultracentrifugation as modified for normal rats[21-22]. Each of the fractions was analyzed for cholesterol, triglyceride, neutral lipids, phospholipid and protein levels.

Enzymatic studies

In vitro enzymatic studies were performed using 10% homogenates of liver mucosa from normolipidemic Sprague Dawley male rats (~280g). The liver homogenates were prepared in 0.25 M sucrose + 0.001 M (ethylenedinitrilo)tetraacetic acid [EDTA], pH 7.2. Acetyl coenzyme A synthetase[23] and adenosine triphosphate dependent citrate lyase activities[24] were determined spectrophotometrically at 540 nm as the hydroxylamate of acetyl coenzyme A formed after 20 min at 37°C. Cholesterol-7α-hydroxylase activity was determined using [1,2-$^{3}$H]cholesterol (60 mCi/mmol)[25], and acyl CoA cholesterol acyl transferase activity was determined using [1-$^{14}$C]oleic acid (56.7 mCi/mmol)[26]. Cholesterol synthesis was measured using [1-$^{14}$C] acetyl CoA (62 mCi/mmol) and a post-mitochondrial supernatant (9000 g x 20 min) which was incubated for 60 min at 37°C [27]. The digitonide derivative of cholesterol was isolated and counted [28]. Cholesterol ester hydrolase activity was determined using 1-$^{14}$C cholesterol oleate [56.6 mCi/mol] [29].

For acetyl coenzyme A carboxylase activity, the enzyme had to be polymerized for 30 min at 37°C and then the assay mixture containing sodium $^{14}$C-bicarbonate (41.0 mCi/mmol) was added and incubated for 30 min at 37°C with test drugs[30]. sn-Glycerol-3-phosphate acyl transferase activity was determined with sn-glycerol-3-phosphate [L-2-3H(N)] (7.1 Ci/mmol) with the microsomal fraction of liver homogenates. The reaction was terminated after 60 min and the lipids were extracted with chloroform/methanol (2:1) containing 1% HCl and counted[31]. Phosphatidylate phosphohydrolase activity was measured as inorganic phosphate released after 60 min[32]. The released inorganic phosphate after color development with ascorbic acid and ammonium molybate was quantitated at 820 nm. Hepatic lipoprotein lipase was determined using glycerol-tri-$^{14}$C-palmitate [64 mCi/mol] emulsified with lecithin by the method of Chait et al.[33]. Protein content of the liver homogenates was determined[20].

Data displayed in Tables 1-5 represent means ± standard deviations. The Student's "t" test was applied between control groups and the individual drug treatment groups using the raw data. The analysis of variance (ANOVA) was applied among test drugs and is reported in the text only.

### RESULTS

Selected carboxy- and cyanoborane adducts of cyclohexylamine and toluidine were shown to be significantly active in the structure activity study in mice at 8 mg/kg/day. In comparison to the standards lovastatin and clofibrate, compounds **1**, **2**, **5**, **7**, **8**, **9**, **10**, **13**, **16**, **17**, and **18** lowered serum cholesterol by 23% at 8 mg/kg/day on day 16. Serum triglyceride level on day 16 were reduced by compound **7**, **8**, **9**, **17** and **18** by 27% at 8 mg/kg/day I.P.

Three compounds **7**, **9** and **18** were selected as being representative compounds to further study in rats their hypolipidemic action [Table 2]. In rats at 8 mg/kg/day, compound **9** lowered serum cholesterol levels 32% after 14 days administration orally, whereas compounds **7** and **18** caused at 24-25% reduction. Nevertheless, these compounds were more potent

**Table 1**: In Vivo Hypolipidemic Activity of Carboxy- and Cyanoborane Adducts of Cyclohexylamine and Toluidine in $CF_1$ Male Mice at 8 mg/kg/day, I.P. for 16 days.

| N=6 | Percent of Control ($\bar{X} \pm S.D.$) | | |
|---|---|---|---|
| Compound | Day 9 Serum Cholesterol | Day 16 Serum Cholesterol | Day 16 Serum Triglyceride |
| 1 | 85±4 | 65±2* | 100±4 |
| 2 | 110±6 | 68±4* | 77±5 |
| 3 | 89±4 | 79±1* | 84±11 |
| 4 | 90±2 | 83±8 | 90±5 |
| 5 | 82±4 | 74±9* | 82±15 |
| 6 | 91±8 | 84±2 | 78±9 |
| 7 | 87±2 | 59±3* | 54±10* |
| 8 | 79±4 | 73±2* | 72±8* |
| 9 | 82±3 | 62±5* | 66±7* |
| 10 | 90±9 | 69±6* | 74±6* |
| 11[a] | 73±3* | --- | --- |
| 12 | 76±6* | 78±4* | 84±5 |
| 13 | 83±3 | 68±6* | 84±6 |
| 14 | 58±3* | 83±3* | 99±5 |
| 15 | 54±5* | 84±1* | 112±9 |
| 16 | 73±2* | 67±4* | 84±14 |
| 17 | 90±2 | 77±5* | 73±14* |
| 18 | 81±3 | 76±4* | 61±15* |
| 19 | 87±3 | 87±3* | 78±5* |
| Control[b] | 100±5[e] | 100±7[f] | 100±11[g] |
| Lovastatin[c] | 85±4 | 82±5* | 86±7 |
| Clofibrate[d] | 87±6 | 78±6* | 75±6* |

[a] Dosed at 4 mg/kg/day, toxic

[b] 1% CMC

[c] Dosed at 8 mg/kg/day

[d] Dosed at 150 mg/kg/day

[e] 125 mg/dL serum cholesterol

[f] 128 mg/dL serum cholesterol

[g] 137 mg/dL serum triglyceride

* $p \leq 0.001$, Student's "t" test than lovastatin at 8 mg/kg/day, gemfibrozil at 90 mg/kg/day or clofibrate at 150 mg/kg/day in lowering serum cholesterol levels after 14 days. Compound 9 also most effectively lowered serum triglyceride levels 29% by day 14; yet compounds 7 and 18 only caused 21% reduction. None of the compounds were as effective as gemfibrozil at 90 mg/kg/day with a 38% reduction of serum triglyceride levels after 14 days. However, the compounds at 8 mg/kg/day were more effective than lovastatin at 8 mg/kg/day and approximately equal to clofibrate at 150 mg/kg/day. Daily food consumption and total body weight of the treated animals were significantly no different than the control group for 14 days.

**Table 2**: <u>In Vivo</u> Hypolipidemic Activity of Carboxy- and Cyanoborane Adducts of Cyclohexylamine and Toluidine in Sprague-Dawley Male Rats at 8mg/kg/day Orally for 14 Days.

| *N*=6 | | Percent of Control ($X \pm S.D.$) | | | |
|---|---|---|---|---|---|
| Compound | Food[a] | Day 7 | | Day 14 | |
| # | Consumption | Cholesterol | Triglyceride | Cholesterol | Triglyceride |
| 7 | 105±6 | 100±9 | 72±7* | 75±10 | 79±7 |
| 9 | 102±7 | 118±6 | 56±4* | 68±8* | 71±7 |
| 18 | 100±7 | 87±9 | 71±4 | 76±7 | 79±4 |
| Control[b] | 100±6 | 100±6[f] | 100±4[g] | 100±6[h] | 100±5[i] |
| Lovastatin[c] | --- | 85±4 | 91±5 | 82±5 | 86±7 |
| Gemfibrozil[d] | 91±5 | 91±5 | --- | 82±7 | 62±5 |
| Clofibrate[e] | --- | 89±7 | 83±6 | 86±5 | 74±7 |

[a] Control = 22.1±1.3 g/day/rat

[b] 1% CMC

[c] Dosed at 8 mg/kg/day

[d] Dosed at 90 mg/kg/day

[e] Dosed at 150 mg/kg/day

[f] 73 mg/dL total serum cholesterol

[g] 75 mg/dL total serum cholesterol

[h] 111 mg/dL serum triglyceride

[i] 112 mg/dL serum triglyceride

* $p \leq 0.001$, Student's "*t*" test

Further tissue lipid extraction studies showed that in rats treated 14 days with compounds 7, 9 or 18 [Table 3], liver, small intestinal mucosa and aorta lipid content was reduced. The triglyceride content was reduced more consistently than the cholesterol content in all three tissues. In the aorta tissue neutral lipids and phospholipid content was also reduced after 14 days treatment. Protein and cholesterol content of the aorta tissue was reduced after treatment with compound 9. Total lipid excretion into the feces was not affected by treatment with the compounds. Fecal triglycerides were elevated significantly by all three compounds; however, phospholipids excretion was reduced [Table 3]. Examination of the lipid content of the rat lipoproteins after 14 days administration showed that all three compounds lowered cholesterol content in the chylomicron and the LDL fraction. The HDL cholesterol content was significantly elevated 2 to 3 fold [Table 4] by all three compounds. Triglyceride content was elevated in the chylomicron fraction after treatment with compounds 9 and 18. Triglyceride content was elevated in the VLDL fraction after treatment with compound 7. HDL triglyceride content was elevated by treatment with compounds 7 and 9. Neutral lipid content of the lipoprotein fractions was not affected by any of the compounds. Phospholipid content of VLDL fraction was reduced by all three compounds while phospholipid content of LDl fraction was

Table 3 *In Vivo* Effects of Carboxy- and Cyanoborane Adducts of Cyclohexylamine and Toluidine on Tissue Lipids in Sprague-Dawley Male Rats After 14 Days at 8 mg/kg/day, Orally

*N*=8 Percent of Control (X ± S.D.)

| | Mg of Lipid Extracted | Cholesterol | Triglycerides | Neutral Lipids | Phospho-lipids | Protein |
|---|---|---|---|---|---|---|
| **Liver** | | | | | | |
| Control | 100 ± 4[a] | 100 ± 5[b] | 100 ± 7[c] | 100 ± 6[d] | 100 ± 7[e] | 100 ± 6[f] |
| 7 | 86 ± 6 | 93 ± 5 | 64 ± 5* | 101 ± 6 | 104 ± 8 | 81 ± 5 |
| 9 | 86 ± 2 | 102 ± 8 | 88 ± 6 | 97 ± 6 | 104 ± 5 | 104 ± 6 |
| 18 | 96 ± 3 | 109 ± 6 | 164 ± 7* | 98 ± 5 | 91 ± 8 | 110 ± 8 |
| **Small Intestine** | | | | | | |
| Control | 100 ± 8[g] | 100 ± 7[h] | 100 ± 6[i] | 100 ± 6[j] | 100 ± 6[k] | 100 ± 7[l] |
| 7 | 76 ± 11* | 94 ± 6 | 55 ± 4* | 105 ± 8 | 93 ± 6 | 117 ± 5 |
| 9 | 93 ± 16 | 117 ± 7 | 67 ± 5* | 98 ± 6 | 79 ± 4* | 115 ± 5 |
| 18 | 88 ± 7 | 112 ± 8 | 87 ± 6 | 91 ± 5 | 66 ± 5* | 119 ± 5 |
| **Aorta** | | | | | | |
| Control | 100 ± 4[m] | 100 ± 5[n] | 100 ± 6[o] | 100 ± 5[p] | 100 ± 6[q] | 100 ± 6[r] |
| 7 | 88 ± 12 | 112 ± 6 | 86 ± 4 | 58 ± 4* | 91 ± 6 | 94 ± 6 |
| 9 | 66 ± 2* | 79 ± 4* | 81 ± 4* | 49 ± 5* | 82 ± 7 | 65 ± 6* |
| 18 | 101 ± 12 | 101 ± 5 | 99 ± 6 | 89 ± 5 | 54 ± 5* | 89 ± 5 |
| **Feces** | | | | | | |
| Control | 100 ± 8[s] | 100 ± 6[t] | 100 ± 6[u] | 100 ± 8[v] | 100 ± 8[w] | 100 ± 6[x] |
| 7 | 99 ± 3 | 87 ± 7 | 148 ± 9* | 99 ± 7 | 57 ± 5* | 104 ± 6 |
| 9 | 101 ± 10 | 95 ± 9 | 155 ± 8* | 97 ± 8 | 65 ± 5* | 115 ± 6 |
| 18 | 104 ± 9 | 87 + 8 | 173 ± 8* | 100 ± 7 | 45 ± 4* | 110 ± 5 |

per gram wet tissue

[a] 50.5 mg lipid
[b] 9.18 mg cholesterol
[c] 6.37 mg triglyceride
[d] 15.70 mg neutral lipid
[e] 27.19 mg phospholipid
[f] 12.02 mg protein
[g] 68.20 mg lipid
[h] 12.02 mg cholesterol
[i] 11.20 mg triglyceride
[j] 16.98 mg neutral lipid
[k] 20.06 mg phospholipid
[l] 42.0 mg protein
[m] 67.5 mg lipid
[n] 5.77 mg cholesterol
[o] 9.85 mg triglyceride
[p] 15.28 mg neutral lipid
[q] 28.8 mg phospholipid
[r] 11.71 mg protein
[s] 11.58 mg lipid
[t] 2.84 mg cholesterol
[u] 1.86 mg triglyceride
[v] 3.39 mg neutral lipid
[w] 5.70 mg phospholipid
[x] 6.99 mg protein
* $p \leq 0.001$, Student's "*t*" test

---

elevated approximately two fold. The protein content of the chylomicron fraction was reduced after 14 day treatment.

The mode of action of the derivatives on rate limiting enzymes involved in liver lipid metabolism showed that the compounds were not HMG-CoA reductase inhibitors. Rather these compounds reduced the activites of cytoplasmic acetyl CoA synthetase and ATP-dependent citrate lyase. Compound 7 and 9 caused a moderate reduction of 11-18% in acyl CoA cholesterol acyl transferase activity. Neutral cholesterol ester

Table 4: In Vivo Effects of Carboxy- and Cyanoborane Adducts of Cyclohexylamine and Toluidine on the Lipid Content of Serum Lipoproteins in Sprague Dawley Male Rats After 14 Days at 8mg/kg/day, Orally

N=4 Percent of Control (X + S.D.)

| | Cholesterol | Triglycerides | Neutral Lipids | Phospholipids | Protein |
|---|---|---|---|---|---|
| **Chylomicron** | | | | | |
| Control | 100 ± 6[a] | 100 ± 7[b] | 100 ± 7[c] | 100 ± 6[d] | 100 ± 5[e] |
| 7 | 67 ± 5* | 105 ± 7 | 92 ± 5 | 86 ± 5 | 79 ± 5 |
| 9 | 67 ± 4* | 158 ± 8* | 96 ± 6 | 85 ± 6 | 81 ± 6 |
| 18 | 50 ± 6* | 136 ± 9* | 91 ± 6 | 104 ± 6 | 80 ± 7 |
| **VLDL** | | | | | |
| Control | 100 ± 4[f] | 100 ± 5[g] | 100 ± 7[h] | 100 ± 7[i] | 100 ± 5[j] |
| 7 | 104 ± 5 | 123 ± 6 | 105 ± 6 | 62 ± 5* | 107 ± 6 |
| 9 | 118 ± 5 | 99 ± 5 | 107 ± 6 | 44 ± 4* | 76 ± 6* |
| 18 | 92 ± 4 | 91 ± 8 | 107 ± 5 | 43 ± 4* | 93 ± 5 |
| **LDL** | | | | | |
| Control | 100 ± 5[k] | 100 ± 6[l] | 100 ± 6[m] | 100 ± 6[n] | 100 ± 8[o] |
| 7 | 65 ± 5* | 114 ± 6 | 98 ± 7 | 202 ± 7* | 89 ± 6 |
| 9 | 50 ± 4* | 101 ± 7 | 100 ± 7 | 182 ± 8* | 133 ± 7* |
| 18 | 63 ± 4* | 110 ± 6 | 99 ± 6 | 214 ± 8* | 96 ± 8 |
| **HDL** | | | | | |
| Control | 100 ± 6[p] | 100 ± 7[q] | 100 ± 6[r] | 100 ± 7[s] | 100 ± 6[t] |
| 7 | 348 ± 10* | 127 ± 5* | 93 ± 4 | 110 ± 7 | 166 ± 6* |
| 9 | 371 ± 9* | 116 ± 6 | 89 ± 5 | 107 ± 6 | 166 ± 6* |
| 18 | 235 ± 9* | 129 ± 6* | 92 ± 6 | 81 ± 7 | 167 ± 5* |

[a] 337 μg cholesterol/mL serum

[b] 420 μg triglyceride/mL serum

[c] 67 μg neutral lipid/mL serum

[d] 149 μg phospholipid/mL serum

[e] 184 μg protein/mL serum

[f] 190 μg cholesterol/mL serum

[g] 22 μg triglyceride/mL serum

[h] 98 μg neutral lipid/mL serum

[i] 26 μg phospholipid/mL serum

[j] 50 μg protein/mL serum

[k] 210 μg cholesterol/mL serum

[l] 45 μg triglyceride/mL serum

[m] 10 μg neutral lipid/mL serum

[n] 41 μg phospholipid/mL serum

[o] 122 μg protein/mL serum

[p] 544 μg cholesterol/mL serum

[q] 630 μg triglyceride/mL serum

[r] 27 μg neutral lipid/mL serum

[s] 153 μg phospholipid/mL serum

[t] 657 μg protein/mL serum

*$p \leq 0.001$, Student's "*t*" test hydrolase activity was reduced by compound 7 by 44%, but the other two compounds only afforded 12-15% reduction. Cholesterol-7α-hydroxylase activity was significantly elevated by all three compounds. Acyl CoA carboxylase activity was suppressed 17% by compound 7 and 29% by compounds 9 and 18. sn-Glycerol-3-phopshate acyl transferase activity was reduced 15% by compound 9 and 19% by compound 18. Phosphatidyate phosphohydrolase activity was reduced markedly 76% by compound 7, 82% by compound 9 and 88% by compound 18. Hepatic lipoprotein lipase activity was reduced 29% by compound 7 and 19% by compound 18.

**Table 5:** In Vitro Inhibtion of Liver Lipid Enzyme Activies by Carboxy- and Cyanoborane Adducts of Cyclohexylamine and Toluidine

Percent of Control ($\bar{X} \pm S.D.$)

| N=6 | | Compound 7 | | |
|---|---|---|---|---|
| Enzyme Assay | Control | 25µM | 50µM | 100µM |
| Acetyl CoA Synthetase | 100±7[a] | 98±7 | 88±6 | 71±6* |
| ATP-dependent Citrate Lyase | 100±5[b] | 92±4 | 73±5* | 67±4* |
| HMG-CoA Reductase | 100±7[c] | 107±6 | 108±7 | 129±7* |
| Acyl CoA Cholesterol Acyl Transferase | 100±6[d] | 122±6 | 84±6 | 82±7 |
| Neutral Cholesterol Ester Hydrolase | 100±5[e] | 71±6 | 66±6* | 56±5* |
| Cholesterol-7-alpha-hydroxylase | 100±6[f] | 122±5* | 125±6* | 150±6* |
| Acyl CoA Carboxylase | 100±5[g] | 92±5 | 84±5 | 83±6 |
| *sn*-Glycerol-3-Phosphate Acyl Transferase | 100±6[h] | 115±6 | 109±6 | 101±5 |
| Phosphatidylate Phosphohydrolase | 100±5[i] | 108±6 | 90±6 | 24±5* |
| Lipoprotein Lipase | 100±6[j] | 117±6 | 102±4 | 71±5* |

| N=6 | | Compound: 9 | | |
|---|---|---|---|---|
| | Control | 25µM | 50µM | 100µM |
| Acetyl CoA Synthetase | 100±7[a] | 99±6 | 84±5 | 80±5* |
| ATP-dependent Citrate Lyase | 100±5[b] | 115±6 | 114±7 | 78±6* |
| HMG CoA Reductase | 100±7[c] | 135±7* | 132±6* | 135±4* |
| Acyl CoA Cholesterol Acyl Transferase | 100±6[d] | 118±5 | 93±4 | 89±5 |
| Neutral Cholesterol Ester Hydrolase | 100±5[e] | 94±6 | 89±4 | 88±6 |
| Cholesterol-7-alpha-hydroxylase | 100±6[f] | 205±8* | 237±8* | 198±7* |
| Acyl CoA Carboxylase | 100±5[g] | 79±5* | 75±4* | 71±5* |
| *sn*-Glycerol-3-Phosphate Acyl Transferase | 100±6[h] | 107±6 | 95±4* | 85±4 |
| Phosphatidylate Phosphohydrolase | 100±5[i] | 46±5* | 31±5* | 18±3* |
| Lipoprotein Lipase | 100±6[j] | 125±5 | 114±4 | 96±4 |

**Table 5 (Continued):**

| N=6 Enzyme Asdsay | Control | Compound: 18 25μM | 50μM | 100μM |
|---|---|---|---|---|
| Acetyl CoA synthetase | 100±7[a] | 104±7 | 96±5 | 66±5* |
| ATP-dependent Citrate Lyase | 100±5[b] | 125±6 | 123±4 | 101±5 |
| HMG CoA Reductase | 100±7[c] | 116±5 | 113±8 | 96±5 |
| Acyl CoA Cholesterol Acyl Transferase | 100±6[d] | 108±7 | 103±4 | 97±6 |
| Neutral Cholesterol Ester Hydrolase | 100±5[e] | 109±5 | 106±4 | 85±6 |
| Cholesterol-7-alpha-hydroxylase | 100±6[f] | 179±6* | 241±5* | 250±6* |
| Acyl CoA Carboxylase | 100±5[g] | 84±3 | 82±6 | 71±4* |
| *sn*-Glycerol-3-Phosphate Acyl Transferase | 100±6[h] | 98±6 | 89±5 | 81±4* |
| Phosphatidylate Phosphohydrolase | 100±5[i] | 24±3* | 17±2* | 12±2* |
| Lipoprotein Lipase | 100±6[j] | 110±6 | 102±5 | 81±4* |

[a] 28.5 mg acetyl CoA formed/g wet tissue [b] 30.5 mg citrate hydrolyzed/g wet tissue [c] 384900 dpm cholesterol formed/g wet tissue [d] 224000 dpm/mg of microsomal protein [e] 56436 dpm/mg wet tissue [f] 4808 dpm/mg of microsomal protein [g] 537800 dpm/mg wet tissue [h] 302010 dpm/mg wet tissue [i] 16.7 μg $P_i$ released/g wet tissue [j] 278538 dpm/g wet tissue
* $p \leq 0.001$, Student's "*t*" test

### DISCUSSION

These studies have shown that carboxy- and cyanoborane adducts of cyclohexylamine and toluidine possessed good hypolipidemic acitivity in rodents. They were approximately equal in their ability to lower serum cholesterol and triglyceride levels in mice at 8 mg/kg/day as the heterocyclic amine boranes. Thus, it appears the nitrogen does not have to be in the aromatic ring to retain good hypolipidemic activity. Like the heterocyclic amine boranes there was no significant difference between carboxyboranes and cyanoboranes as far as their ability to lower serum cholesterol and triglycerides after 16 days administration I.P. in mice. However, it should be noted that compound 7 did not afford as good activity in rats as it did in mice, although its magnitude of serum lipid reduction was still comparable to the standards lovastatin and clofibrate. This type of species variation has been noted before with boron derivatives[7]. Like many of the boron derivative these new compounds lowered cholesterol, triglyceride and phospholipid content in the major organs. What is most important is the fact that compound 9 was very effective in lowering cholesterol content within the rat aorta wall after 14 days administration. These adducts were very impressive with regard to their effects on lipoprotein lipid content. An ideal hypolipidemic agent should lower cholesterol content of the VLDL and LDL fraction since these lipoproteins are responsible for conducting cholesterol to the peripheral tissue including the arterial walls, whereas HDL cholesterol content should be high so that cholesterol may be conducted back to the liver for biliary excretion. These derivatives were able to successfully lower VLDL and LDL cholesterol content and elevate HDL cholesterol content. In fact the elevation HDL cholesterol content afforded by these agents is much higher than the standards lovastatin with a 29% increase [34] or gemfibrozil with a 104% increase in rat HDL-cholesterol content[11]. Supposedly, a clinical agent which lowers LDL- and VLDL-cholesterol content but elevates HDL-cholesterol content protects man from mycocardial infarction[35].

The mode of action of the derivatives on regulatory enzyme activities involved in lipid metabolism appeared to be at site which regulated cytoplasmic formation of acetyl-CoA for cholesterol and fatty acid synthesis. These agents were not HMG-CoA reductase inhibitors whereas the heterocyclic amine-carboxy- and cyanoboranes were potent inhibitors. The amine-borane adducts of cyclohexylamine and toluidine accelerated cholesterol-7α-hydroxylase activity more than the heterocyclic amine boranes. This would suggest that the former compounds probably accelerate the conversion of cholesterol to bile acids theoretically increasing cholesterol clearance from the body. These derivative marginally inhibited acyl-cholesterol acyl transferase activity which should reduce cholesterol ester storage in tissues. In aorta walls this enzyme plays a role in depositing cholesterol esters in foam cells and increased plaque growth; thus inhibition of its activity should slow the process. It was hoped that the agents would accelerate cholesterol ester hydrolase activity. This enzyme breaks cholesterol esters down to free cholesterol which can be picked up by HDL to be conducted back to the liver via the reverse cholesterol transport mechanism for biliary excetion of cholesterol. This enzyme level is low in man but it is inducable by selective hypolipidemic agents to cause more clearnce of aorta wall lipids[36]. The heterocyclic amine-boranes were able to increase the activity of cholesterol ester hydrolase.

The neutral lipid and triglyceride pathways were inhibited by these derivatives at the site of phophatidylate phosphohydrolase, so that triglyceride can not be formed from phospholipids. The other regular enzyme of the triglyceride pathway, sn-glycerol-3-phosphate acyl transferase, was marginally inhibited by compounds 9 and 18. These adducts were more potent in this respect than the heterocyclic amine-boranes. Since hepatic lipoprotein lipase activity was also inhibited by the amine borane adducts of cyclohexylamine and toluidine, the inhibition of these three enzyme activities should be of a magnitude to lower triglyceride levels in the blood and tissue. Furthermore, some accelerated loss of triglycerides occurred in the feces, although bile excretion of triglycerides is considered to be of a small magnitude under normal conditions.

In conclusion, the amine-borane adducts of cyclohexylamine and toluidine maintain good hypolipidemic activity and achieve alteration in lipid metabolism and clearance which are desirable. Further investigation is warrented to evaluate such derivatives for clinical trails.

### REFERENCES

1. Hall, I.H., Spielvogel, B.F., Das, M.K., and McPhail, A.T. (1981) J. Pharm. Sci. 70, 339.
2. Hall, I.H., Das, M.K., Harchelroad, Jr., F., Wisian-Neilson, P., McPhail, A.T. and Spielvogel, B.F., (1981) J. Pharm. Sci. **70**, 339.
3. Sood, A., Sood, C.K., Spielvogel, B.F., Hall, I.H., and Wong, O.T., (1991) Arch Pharm. 324, 423.

4. Hall, I.H., Spielvogel, B.F., Sood, A., Ahmed, F., and Jafri, S. (1987) J. Pharm. Sci. **76**, 359.
5. Hall, I.H., Chen, S.Y., Rajendran, K.G., Sood, A., Spielvogel, B.F., and Shih, J. (1994) Environ. Health Perspect **102**, 21.
6. Sood, A., Sood, C.K., Spielvogel, B.F., and Hall, I.H. (1990) European. J. Med. Chem. **25**, 301.
7. Hall, I.H., Sood, A., and Spielvogel, B.F., (1991) Biomed. Pharmacother **45**, 333.
8. Hall, I.H., Brotherton, R.J.,Griffin, T.S., Sood, A., and Spielvogel, B.F. (1991) Biomed. Biochim. Acta **50**, 1007.
9. Sood, A., Sood, C.K., Spielvogel, B.F., Hall, I.H., and Wong, O.T (1992) J. Pharm. Sci. **81**, 458.
10. Hall. I.H., Wong, O.T., Sood, A. Spielvogel, B.F., and Morse, K.W. (1992) Pharmacol. Res. Commun. **25**, 259.
11. Hall, I.H., Burnham, B.S., Shaw, B.R., Rajendran, K.G., Chen, S.Y., Sood, A., and Spielvogel, B.F. (1993) Biomed. Pharmacotherapy **47**, 79.
12. Hall, I.H., Reynolds, D.J., Chang, J., Spielvogel, B.F., Griffin, T.S. and Docks, E.L. (1991) Arch. Pharm. **324**, 573.
13. Burnham, B.S., Chen, S.Y., Sood, A., Spielvogel, B.F., and Hall, I.H. (1995) die Pharmazie, submitted.
14.. Ness, A.T., Pastewka, J.V., and Peacock, A.C. (1959) Clin. Chem. Acta. **10**, 229.
15. Hall, I.H., Cocolas, G.H., and Voorstad, P.J. (1984) J. Pharm. Sci. **73**, 812.
16. Bligh, E.G., and Dyer, W.J. (1959) J. Biochem. Physiol. **37**, 911.
17. Folch, J., Lees, M., and Stanley, G.H.C. (1957) J. Biol. Chem. **226**, 497.
18. Bragdon, J.H. (1951) J. Biol. Chem. **190**, 513.
19. Stewart, C.P., and Hendry, E.G. (1935) J. Biochem. **29**, 1688.
20. Lowry, O.H., Rosebrough, N.J., Farr, A.L., and Randall, R.J. (1951) J. Biol. Chem. **193**, 263.
21. Havel, R.L., Eder, H.A., and Bragdon, J.M. (1955) J. Clin. Invest. **34**, 1345.
22. Mookerjea, E.S., Parks, C.E., and Kuksis, A. (1975) Lipids **10**, 374.
23. Hoffmman, M., Weiss, L., and Wieland, O.H. (1978) Anal. Biochem. **84**, 441.
24. Goodridge, A.G.(1973) J. Biol. Chem. **248**, 4318.
25. Haven, G.T., Krzemian, J.R., and Nguyen, T.T. (1973) Res. Commun. Chem. Path. Pharmacol. **6**, 253.
26. Wada, F., Hirata, K., and Sakameto, Y. (1989) J. Biochem. **65**, 171.
27.Shefer, S., Hauser, S., and Mosbach, E.H. (1978) J. Lipid Res. **9**, 328.
28. Balasubramaniam, S., Mitropoulos, K.A., and Venkatesam, S. (1978) European J. Biochem. **90**, 377.
29. Hall, I.H., Wong, O.T., and Wyrick,S.D. (1988) Pharm. Res. **5**, 413.
30. Greenspan, M.D., and Lowenstein, J.M. (1968) J. Biol. Chem. **243**, 6273.
31. Lamb, R.G., Wyrick, S.D., and Piantadosi, C. (1977) Athersclerosis **27**, 147.
32. Mavis, R.D., Jacob, N., Finkelstein, J.N., and Hall, B.P. (1978) J. Lipid Res. **19**, 467.
33. Chait, A., Iverius, P.H., and Brunzell, J.D. (1982) J. Clin. Invest. **69**, 490.

34. Hall, I.H., Wong, O T., Reynolds, D.J., and Simlot, R. (1993) J. Pharm. Sci. 82, 565.
35. Miettinen, T.A., Huttunen, J.K., Strandberg, T., Naukkarinen, V., Mattila, S., and Kumlin T. (1981) Lancet 2, 478.
36. Wong, O.T., Williams, Jr. W.L., Oswald, B.S., and Hall. I.H. (1992) Res. Commun. Chem. Path. and Pharm. 76, 3.

**Received: April 7, 1995 - Accepted: May 5, 1995 - Received camera-ready revised version: May 23, 1995**

